# Quality of Life among Patients with Glaucoma in the West Bank, Palestine: A Cross-Sectional Study

**DOI:** 10.4314/ejhs.v33i4.4

**Published:** 2023-07

**Authors:** Wejdan Khatib, Raj'a Zyoud

**Affiliations:** 1 Faculty of Graduate Studies, Arab American University-Palestine, West Bank-Palestine; 2 Nursing Faculty, Arab American University-Palestine, West Bank-Palestine

**Keywords:** Quality of Life, Glaucoma, Health Survey (SF-36)

## Abstract

**Background:**

Glaucoma is a serious eye disease that impairs eyesight and negatively impacts quality of life. If left untreated, glaucoma can lead to blindness. This study aims at assessing the quality of life among glaucoma patients in the West Bank (WB) of Palestine and main influencing the factors.

**Methods:**

This is a cross-sectional study and included two questionnaires; the Short-Form 36 Health Survey (SF-36) and Glaucoma Quality of Life-15 (GQL-15). Data were collected from a systematically randomized 100 glaucoma patients in the WB. ANOVA test was used to compare means of continuous variables at a statistically significant P value ≤ to 0.05.

**Results:**

The overall quality of life among glaucoma patients was relatively suboptimal. The analysis revealed that the glaucoma quality of life is worse among older patients (mean=3.55±0.64), patients who are less educated (mean=3.91±0.77), among patients who were unemployed (mean=3.44±0.86), and patients who were treated in private clinics (3.57±0.8). Patients with good health (mean=2.48±0.94), type of glaucoma (close glaucoma; mean=3.22±0.9), less than 5 years duration of glaucoma (mean=2.88±1.13), and less than 5 years duration of cryonic diseases (mean=2.48±0.73 have a better glaucoma quality of life.

**Conclusions:**

This study revealed numerous factors that can impact the quality of life of glaucoma patients in WB. Health professionals, specialists, ophthalmologists, and health educators should be aware of how various socioeconomic and general health factors impact the quality of life of glaucoma patients in order to better diagnose, manage, guide, and educate patients for better health outcomes.

## Introduction

Glaucoma is a group of progressive optic neuropathies characterized by a degeneration of retinal ganglion cells and retinal nerve fiber layers that result in changes in the optical nerve head (1). Glaucoma is associated with intraocular pressure (IOP)-related damage to the optic nerve, which results in the loss of retinal ganglion cells (2). Globally, over 80 million individuals are estimated to be impacted by glaucoma, which is the main cause of permanent blindness. By 2040, this number is projected to reach more than 110 million(3). Age and frailty, gender, myopia, genetics, family history, smoking, race, systemic hypotension and hypertension, vasospasm, use of systemic or topical steroids, migraine, obstructive sleep apnea syndrome, and most significantly, increased IOP are all risk factors (4-6). According to previous studies, almost half of glaucoma cases are estimated to be undiagnosed (7). Glaucoma can be classified as either open-angle or angle-closure glaucoma according morphology of the anterior chamber. The global prevalence of primary open-angle glaucoma is about 3.1, which represents six times greater than a global prevalence of the primary angle-closure glaucoma(8). The primary open-angle glaucoma was more common (4.2%) in Africa, whereas primary angle-closure glaucoma was more common (1.1%) in Asia (8).

The glaucoma impacts have been assessed by various measures. The Quality of life (QoL) is one of those indicators because it offers information about the nature of the condition and patients' experiences, as well as acting as a measure for therapy effectiveness. It can also be used as a measure of the effectiveness of a medical intervention. Assessing QoL in undiagnosed glaucoma patients can also be used to detect cases early and as a result, achieve better outcomes. Additionally, QoL examines how glaucoma affects the patient as a whole and can be used to monitor glaucoma patients' progress. QoL reflects the individual's overall wellbeing and covers arrears of physical, mental, general, and social health and functioning (9). QoL measures have increased in use in healthcare over the past few years and have become major goals of treatment (10). The primary factor contributing to the decline in QoL is the loss of visual abilities, which makes it more difficult to walk, drive, read, and see to the side. Following the demanding treatment plans has an impact on QoL as well (11). The fear of blindness is itself debilitating. Social withdrawal is one aspect that affects QoL of glaucoma patients. The financial, medical, and social problems are not only borne by the patient but the family as well(12). Patients' QoL may differ based on their views on the disease, their cultural and environmental contexts (13).

Data on the prevalence of glaucoma are not available in Palestine. One study examined the QoL of glaucoma patients in Gaza (14) but the needs and obstacles of glaucoma patients in the West Bank have not been studied. Therefore, this study aims to assess the QoL of glaucoma patients in the West Bank and explore the main influencing factors.

## Materials and Methods

**Study design**: This is a descriptive, analytical, cross-sectional study. The cross-sectional design was selected as it was judged to be the most appropriate method to fulfill the aim of the study in a limited time and money.

**Study setting and population**: The study was conducted at WB in either governmental or private “non-governmental” ophthalmic clinics or hospitals. The study recruited glaucoma patients from 4 private hospitals and 4 governmental hospitals. The private hospitals are An-Najah national university hospital, St John eye hospital, Surgi-Care center, and Alrazi hospital. The governmental hospitals are Alia hospital, Hugo Chaves Ophthalmic hospital, Rafidia hospital, and Palestinian medical complex. The study population was all patients with glaucoma in WB. All registered glaucoma patients with no other ocular comorbidity were estimated to be about (200).

**Sampling and sample size**: Study sampling is a systematic random sample to select every other patient. All the registered glaucoma patients were grouped in a list and then systematically selected (sampling interval was 2). The calculated sample size using the G*Power software was 100 patients with glaucoma. The study sample size was 100 with a 100% response rate.

**Inclusion criteria**: Adult glaucoma patients (18 years old and above) who were able and willing to answer the questions in the questionnaire who were diagnosed of glaucoma more than 6 months of this study and patients who were on medical therapy were included in the study.

**Exclusion criteria**: Patients with any ocular condition that could impair vision such as cataract that is clinically diagnosed, macular degeneration, or any other ophthalmic condition were excluded. In addition, patients who had incisional ocular surgery, incisional glaucoma, cataract surgery, or previously treatment by laser and patients who did not understand the questions or were not willing to answer them were not recruited into the study.

**Study instrument**: The questionnaires were developed after reviewing previous studies dealing with the similar patients. Some questions were gathered and modified from other questionnaires of similar published studies (15-18). The study included two questionnaires; the Short-Form 36 Health Survey (SF-36) and Glaucoma Quality of Life-15 (GQL-15). The Short-Form 36 Health Survey (SF-36) questionnaire focuses on the participant's experiences, feelings, beliefs, perceptions and convictions concerning their health-related quality of life. It consists of closed-ended structured questions. These questions are particularly related to the eight quality of life indicators which are (General health, Emotional roles limitation, Physical functioning, social functioning, Physical roles limitation, Mental health, Vitality and Bodily pain). The validity and reliability of this questionnaire was confirmed in numerous studies(19, 20). The Cronbach's alpha in most studies was above (0.7). The Glaucoma Quality of Life-15 (GQL-15) questionnaire is concise, easy to administer and considered one of the better glaucoma-specific instruments, with good acceptability among clinicians and patients. It asks 15 rating-scored questions to assess the degree of functional disability caused by glaucoma. The questions include six questions related to peripheral vision, six related to dark adaptation and glare, two related to central and near vision and one related to outdoor mobility. Responses are coded on a 5-point Likert scale ranging from 1) no difficulty to 5) severe difficulty. The subscale score for each factor is calculated as the average of the sum of the item scores. Higher subscale scores imply lower QoL. Score less than 50% was assumed to indicate poor QoL. All questionnaire items were translated into Arabic (the mother tongue of participants). Each item of the English and Arabic versions was grouped together and validated by experts with health and research backgrounds.

**Validity and Reliability of the questionnaires**: Face and content validity were ensured through group of experts who reviewed and commented on the questions. Feedback was obtained from experts and modification was done accordingly. Piloting among 10 glaucoma patient was done. As a result of the participants' perception that the questionnaire was clear and uncomplicated, they were included in the actual research. The overall Cronbach's alpha for both questionnaires equals 0.902; which indicates good reliability of the entire questionnaire. The values of Chronbach's Alpha for the questionnaire domains ranged from 0.769 to 0.955.

**Data collection and analysis**: The researcher started data collection by introducing herself to the participants and presented full directions and clarification about the study, its goals, and the significance of providing accurate answers. The data collection was taking place at suitable place and convenient time, with adherence to all ethical considerations. Self-administered questionnaire was used to gather the data. The researcher helped the patients and wrote down the answers of the patients who were unable to write down their answers due to their inability to see well. Data collection took place in the period between August, 2021 to December 2021. The researcher used Statistical Package of Social Science (SPSS-version 25) program for data entry and analysis. Descriptive and inferential analyses were done. The t-test and ANOVA were used to compare the total mean score of QoL and sociodemographic variable and general health variables. On the other hand, the Pearson test was used to check the coloration between the total mean score of QoL and total mean scores of the main dimensions of the Short-Form 36 Health Survey (SF-36). P-value less than or equal to 0.05 was considered statistically significant.

**Ethics approval**: Ethical approval was obtained from the Arab American University-Palestine (IRB.028/2021). Also, a permission letter (Research ethics committee approval) from the Palestinian Ministry of Health (EA339-2021) was also obtained to allow the researcher to collect data. Verbal informed consent was obtained from all participants.

## Results

Table 1 shows that more than half of the participants were males (52.0%). The majority of participants (71%) were above 51 years old. About half of participants (53%) were well educated who had bachelor degree and above. Most of participants (75%) were unemployed and (61%) had a monthly salary less than 2000 New Israeli Shekel (NIS).

**Table 1:** Distribution of the study population according to socio-demographic data (N= 100)

Variables	Categories	n	%
Gender	Male	52	52.0
	Female	48	48.0
Years in age (years)	18 - 50	29	29.0
	51-60	35	35.0
	More than 60	36	36.0
Educational Level	Illiterate	24	24.0
	Primary level	14	14.0
	Preparatory level	9	9.0
	University level	25	25.0
	Higher education	28	28.0
Marital status	Unmarried	10	10.0
	Married	90	90.0
Religion	Muslim	93	93.0
	Christian	7	7.0
North districts	Nablus	12	21.8
	Tulkarem	10	18.2
	Jenin	12	21.8
	Tubas	7	12.7
	Salfit	9	16.4
	Qalqilya	5	9.1
Health Facility of care	Hugo Chavez Ophthalmic		
	Hospital	35	35.0
	St. Joseph Hospital	2	2.0
	Private Clinic	63	63.0
Occupation	Work	25	25.0
	Unemployed	75	75.0
Monthly income (NIS)	Less than 1000 NIS	18	18.0
	1000 – 2000 NIS	43	43.0
	More than 2000 NIS	39	39.0

NIS: New Israeli Shekel

The overall glaucoma quality of life was relatively suboptimal with percent mean 66%. Table 2 shows that the worst QoL items are difficulties in reading newspaper (% mean=75), adjusting to bright lights (% mean=74), and walking after dark (% mean=73). The items with least difficulty are judging distance of foot to step/curb (% mean=57), recognizing faces (% mean=58), and crossing the road (% mean=58).

**Table 2:** Distribution of the participants according to responses about glaucoma quality of life (N= 100)

Glaucoma Quality of Life	No difficulty	A little bit of difficulty	Some difficulty	Quite a lot of difficulty	Large difficulty	Not done at all due to vision problems	Mean	SD	% Mean	Rank

	Percent									
Reading newspapers	4	3	8	22	30	33	*3.7*	0.5	75	1
Walking after dark	4	4	6	22	36	28	*3.7*	1.3	73	3
Seeing at night	5	5	5	20	38	27	*3.6*	1.3	72	4
Walking on uneven Ground	4	5	11	30	30	20	*3.4*	1.3	67	7
Adjusting to bright Lights	3	0	4	31	43	19	*3.7*	1.0	74	2
Adjusting to dim Lights	1	0	10	34	39	16	*3.6*	0.9	72	5
Going from light to dark room or vice versa	2	2	7	32	42	15	*3.6*	1.0	71	6
Tripping over objects	3	8	9	41	30	9	*3.1*	1.2	63	8
Seeing objects coming from the side	5	6	13	35	29	12	*3.1*	1.3	63	9
Crossing the road	3	9	19	41	18	10	*2.9*	1.1	58	13
Walking on steps /stairs	3	9	15	39	23	11	*3.0*	1.2	61	10
Bumping into Objects	3	7	22	39	19	10	*2.9*	1.2	59	11
Judging distance of foot to step/curb	2	9	30	30	19	10	*2.9*	1.2	57	15
Finding dropped Objects	5	10	15	39	19	12	*2.9*	1.3	59	12
Recognizing faces	3	10	21	36	18	12	*2.9*	1.2	58	13
**Total**							***3.3***	**0.9**	**66.0**	

Table 3 shows the association between glaucoma QoL and main influencing factors. Pearson correlation showed that there is a positive significant association between the glaucoma QoL and all influencing factors as information about daily activities, information about problems as a result of physical health, information about problems as a result of emotional problems, information about feelings and how things have been with you during the past 4 weeks, information about truthiness and fault of specific statements (P < 0.05). This indicates that better QoL is associated with better daily activities, physical health, emotional health, feelings, and truthiness.

**Table 3:** Correlation between glaucoma quality of life and main influencing factors (SF-36) among the study population (N= 100)

Item	% Mean	r	P-value
Information about daily activities	63.3	0.385	0.000
Information about problems as a result of Physical Health	78.0	0.289	0.004
Information about problems as a result of Emotional Problems	80.0	0.408	0.000
Information about feelings and how things have been with you during the past 4 weeks	65.5	0.257	0.010
Information about truthiness and fault of specific statements	58.0	0.319	0.001

(r) = represents Pearson coloration coefficient

The relation between glaucoma QoL and sociodemographic data is shown in Table 4. ANOVA was used to compare the mean of the 15-item glaucoma QoL scale among background variables. The results reveal that socio-demographic factors that influence QoL for patients with glaucoma are age, educational level, type of health facility, and occupation (P<0.05). Glaucoma patients with higher age (more than 60 years old) had lower QoL (p = 0.047). Glaucoma patients' QoL is much better in St. Joseph hospital (1.9) compared to Hugo Chavez Ophthalmic Hospitals (2.81) and worst in private clinic (3.57). The glaucoma QoL is better among patients who work than among patients who do not work (2.76 vs 3.44 respectively), probably because people who work are younger and in a better health status; and also, QoL is better among the educated patients.

**Table 4:** The relation between glaucoma quality of life and socio-demographic data (N=100)

Variables	Categories	n	Mean ±SD	t/F	P-value
Gender	Male	52	3.21±1.02	-0.645	0.520
	Female	48	3.33±0.88		
Age	50 or less	29	2.97±1.1	3.164	0.047*
	51-60	35	3.22±1.03		
	More than 60	36	3.55±0.64		
Educational Level	Illiterate	24	3.91±0.77	4.965	0.001*
	Primary level	14	3.41±0.89		
	Preparatory level	9	2.9±0.88		
	University level	25	2.91±0.94		
	Higher education	28	3.08±0.92		
Marital status	Unmarried	10	3.09±1.2	-0.635	0.527
	Married	90	3.29±0.92		
Religion	Muslim	93	3.23±0.97	-1.322	0.189
	Christian	7	3.72±0.48		
North districts	Nablus	12	3.3±1.02	1.317	0.272
	Tulkarem	10	2.99±0.79		
	Jenin	12	3.62±0.7		
	Tubas	7	4.03±0.78		
	Salfit	9	3.5±1.11		
	Qalqilya	5	3.3±1.02		
Employment	Work	25	2.76±1.04	-3.232	0.002*
	Did not work	75	3.44±0.86		
Monthly income (NIS)	Less than 1000	18	3.45±0.85	0.555	0.576
	1000 – 2000	43	3.28±1.06		
	More than 2000	39	3.17±0.87		
Type of Health Facility	Hugo Chavez Ophthalmic Hospital	35	2.81±0.89	11.323	0.000*
	St. Joseph hospital	2	1.9±2.5		
	Private Clinic	63	3.57±0.8		

* P-value<0.05 indicate significant differences, NIS: New Israeli Shekel

The relation between glaucoma QoL and life and general health data shown is in Table 5 and Table 6. ANOVA test was used to compare the mean of the 15-item glaucoma QoL scale among general health variables. The results reveal that general health data factors that influence QoL for patients with glaucoma were general health, type of glaucoma, duration of glaucoma disease, and duration of chronic disease (P<0.05).

**Table 5:** The relation between glaucoma quality of life and general health variables data (N= 100)

General Health Information	Categories	n	Mean±SD	t/F	P-value
In general, would you say your health	Excellent	7	3.1±0.74	4.078	0.004*
	Very good	10	2.48±0.94		
	Good	71	3.42±0.92		
	Fair	8	2.68±0.86		
	Poor	4	4.02±0.42		
Compared to one year ago, how would you rate your health in general now?	Much better now than one year ago	1	2	3.912	0.006*
	Somewhat better now than one year ago	10	2.35±1.26		
	About the same	76	3.43±0.8		
	Somewhat worse now than one year ago	11	3.19±1.18		
	Much worse now than one year ago	2	2.73±0.57		

* P-value<0.05 indicate significant differences

**Table 6:** The relation between glaucoma quality of life and general health variables data (N= 100)

General Health Information	Categories	n	Mean±SD	t/F	P-value
Did you check your eyes during the medical screening?	Yes	76	3.32±1.02	0.928	0.356
	No	24	3.11±0.67		
Type of Glaucoma	Open Angle	84	3.31±0.91	6.138	0.003*
	Closed Glaucoma	15	3.22±0.9		
	Congenital (since birth)	1	0.13		
Duration of Glaucoma disease	Less than 5 years	44	2.88±1.13	7.568	0.001*
	5 - 10 years	45	3.52±0.63		
	More than 10 years	11	3.76±0.72		
Have you ever been treated and for chronic disease conditions?	Yes	61	3.3±0.79	0.470	0.639
	No	39	3.21±1.16		
Specify	Asthma	3	3.67±1.05	0.455	0.808
	Sickle Cell disease	2	3.67±0.94		
	Diabetes	34	3.28±0.89		
	Hypertension	13	3.15±0.71		
	Cancer	8	3.5±0.34		
	Others	1	2.87		
Duration of Chronic Disease	Less than 5 years	8	2.48±0.73	6.332	0.003*
	10 years	28	3.33±0.68		
	More than 10 years	25	3.54±0.79		
8. Is there any family member with/ history of any of the diseases mentioned above?	Yes	28	3.06±0.89	-1.359	0.177
	No	72	3.35±0.97		

* P-value<0.05 indicate significant differences

## Discussion

To the best of our knowledge, this is the first study conducted among glaucoma patients in order to assess their QoL in the West bank using the GQL-15 and the SF-36 together. However, similar study was performed in the Gaza Strip(14). Despite 71% of participants prescribed their general health as good, the overall glaucoma QoL was relatively suboptimal (66%). On the other hand , the QoL of glaucoma patients was at medium level in Gaza Strip (14) and this is supported by previous findings (21) and whom used SF-36 (22). In another hand, Goldberg and his colleagues (23)and Naveen, et al. (17) used the GQL-15 and ended with similar findings. Jain and his colleagues (24)have used the World Health Organization QoL Brief (WHOQOL-Brief) among 100 patients with glaucoma and found that the QoL was low. The QoL could be attributed to many factors including, but not limited to, visual impairment, side effect of treatments, cost of therapies and inconvenience. Patients with peripheral and central visual impairment are unable to move around, practice daily activities or find objects, and adapting to light changing. Thus, they are potentially at higher risk to falls and accidents. Additionally, it has been observed that the QoL among glaucoma patients' decreases over time as their visual field worsens and their centeral vision field becomes less sensitive (25). Consistent with previous studies, the mean score of GQL-15 is low for central vision and adaptation to dark dimensions. This is exactly in line with findings of previous studies (14), (26) and (17) whom revealed that visual impairment is linked to dark adaptation or glare, like walking after dark, seeing at night and adjusting to different levels of illumination. Dhawan and his colleagues (15) found poor QoL among patients with mild, moderate and severe glaucoma compared to control healthy group and the QoL declined in patients who experienced severe visual loss. Similarly, Onakoya and his colleagues revealed that QoL diminished in every stage of the diseases especially in patients with primary open angle glaucoma(27).

Participants in the age group above 60 years reported significant lower QoL. This finding is consistent with results of Gupta et al. (28) and Béchetoille et al. (29)whom reported negative correlation between age and QoL in the general population. Similarly, Mushtaha and Aljedi (14) revealed low QoL with progression of age. The most reported problems associated with aging are decreased vision, bad reading, walking on stairs and/or identifying persons. This finding has been also proven two decades ago through Salisbury Eye Evaluation project, showed that aging contributes to declining of functional status including the eye(30). Sesar et al. (2020) found contrary results. Indeed, age is linked to physical domain of QoL and human body is negatively affected by complexity of disease, nutritional and emotional status(18). Thus, our finding could be explained by variations of priorities determined the QoL by different age groups and differences of life perception between elderly and middle age population. Lester and Zingirian (2002), however, found no significant relationship between GQL-15 scores and age (31).

Patients who are illiterate or completed primary school had significant low QoL compared to patients with higher education degree. This is consistent to results obtained by Sesar et al. (2020) who reported better QoL among patients completed higher education level(18). This is also confirmed by many studies which revealed significant impact and positive correlation with QoL(27, 28). Indeed, our finding is not surprising, however, because usually individuals with primary education are less committed and adhered to therapeutic regimen as well as less aware about the glaucoma and management practices. Educational level is an important and significant contributing factor that is positively linked to QoL in glaucoma patients (28). Low educated patients demonstrated higher need for information related disease with regard to support for visual impairment, characteristics of the diseases, optimal management and practices (32).

The study revealed a significant difference between patients who were working and those who weren't. Patients who were working had considerably better QoL. This is consistent with result obtained by Khorrami-Nejad et al. (2016) whom reported significant correlation between employment status and QoL(33). In return, Amini, Haghani and Masoumi (2010) demonstrated no significant correlation(34). It could be argued that employed patients are at least able to buy necessary medicines that are not available in governmental or UNRWA clinics. Furthermore, it can be explained by the proportion of patients who sought out private eye clinics. Financial protection and employment are shown to have a great impact on QoL. Therefore, government and other interested stakeholders have to work sincerely toward ensuring social and financial independency of glaucoma patients and ensure suitable work that maintain a respectful life with satisfactory QoL.

Interestingly, the study showed a significantly higher QoL among patient treated in specialized eye hospitals than in private clinics. Such finding is consistent with a previous study concluded that the impact on QoL was higher in patients from the public facilities compared to those from private clinics using the NEI-VFQ questionnaire(35). However, this might indicate how the specialized eye hospitals focus on the technical quality, continuous follow-up, and health education.

This study concluded that age, educational level, and employment are the main influencing factors that can impact the QoL of glaucoma patients in West Bank. Furthermore, the study revealed that better QoL is associated with better daily activities, physical health, emotional health, feelings, and truthiness. It is worth pointing out that the analysis showed that a significant association between the score of QoL and general health, type of glaucoma, duration of glaucoma disease, and duration of chronic disease. Health professionals, specialists, ophthalmologists, and health educators should be aware of how various socioeconomic and general health factors impact the quality of life of glaucoma patients in order to better diagnose, manage, guide, and educate patients for better health outcomes.

This study has limitations. Sample size is small and the researchers recommend replicating the study with a larger sample. The study design is a cross-sectional which limits causal inference. A potential limitation of our study is the reliance on self-reported data, which may lead to recall bias and social desirability bias.
